# Macrophage migration inhibitory factor is overexpressed in pancreatic cancer tissues and impairs insulin secretion function of β-cell

**DOI:** 10.1186/1479-5876-12-92

**Published:** 2014-04-07

**Authors:** Langping Tan, Xiao Ye, Yu Zhou, Min Yu, Zhiqiang Fu, Ruiwan Chen, Baoxiong Zhuang, Bing Zeng, Huilin Ye, Wenchao Gao, Qing Lin, Zhihua Li, Quanbo Zhou, Rufu Chen

**Affiliations:** 1 Department of Pancreaticobiliary Surgery, The Second Affiliated Hospital of Sun Yat-sen University (Sun Yat-sen Memorial Hospital), Sun Yat-sen University, 107 Yan-Jiang Xi Road, Guangzhou 510120, China; 2 Department of Medical Oncology, Sun Yat-sen Memorial Hospital, Sun Yat-sen University, Guangzhou, China; 3 Department of Radiotherapy, The First Affiliated Hospital of Sun Yat-sen University, Guangzhou, China; 4 Department of Emergency Department, Sun Yat-sen Memorial Hospital, Sun Yat-sen University, Guangzhou, China

**Keywords:** Pancreatic cancer, Diabetes mellitus, Macrophage Migration Inhibitory Factor, Biomarker, Diagnosis

## Abstract

**Background:**

Understanding the pathogenic mechanism of pancreatic cancer associated diabetes (PCDM) might help yield biomarkers for the early diagnosis of pancreatic cancer (PC) from population with new-onset diabetes. In the current study, we sought to determine the role of macrophage migration inhibitory factor (MIF) in PCDM pathogenesis.

**Methods:**

The protein and mRNA levels of MIF in paraffin-embedded human PC samples, chronic pancreatitis specimens, and normal pancreas were measured by immunohistochemistry and quantitative reverse-transcriptase polymerase chain reaction. We measured serum levels of MIF in PC patients and controls. The biologic impacts of MIF overexpression on insulin secretion function of mice islets and β cells (HIT-T15) were investigated in vitro.

**Results:**

MIF expression was significantly increased in pancreatic cancer tissues compared with chronic pancreatitis or normal pancreas specimens. The insulin secretion function of both islets and HIT-T15 cells was impaired by indirect co-cultured with PC cells or treated with conditioned media from them. Stable MIF knock-down significantly decreased the diabetogenic effect of PC cells, while MIF knock-in HPDE6 cells demonstrated a strong inhibitory effect on insulin secretion function of islets and HIT-T15 cells. MIF impaired βcell function by depressing the Ca^2+^ currents, decreasing L-type Ca^2+^ channel α1 subunit protein expression level, and enhancing p-Src activity. Mean serum level of MIF was significant higher in new-onset diabetes associated PC patients in comparison with other groups.

**Conclusions:**

MIF is up-regulated in patients with pancreatic cancer and causes dysfunction of insulin secretion in β-cells.

## Background

Pancreatic cancer (PC) is a highly malignant neoplasm and the forth-leading cause of cancer death. The 5-year survival of PDAC is only about 5%, and this figure has remained nearly unchanged over the past two decades [1,2]. Highlighting its dismal prognosis, principally because the cancer-specific symptoms occur late, and a screening strategy for asymptomatic patients of sporadic pancreatic cancer has not been established [3,4]. Thus, the discovery of valuable biomarkers for early diagnosis PC will be of great significance.

The relationship between diabetes mellitus (DM) and PC has long been studied for decades, but it became more interesting since the existence of a bidirectional association between the two entities was discovered: the risk of pancreatic cancer is high with new-onset diabetes (5–8-fold) whereas the risk levels out at about 1.5-fold in long-standing diabetes patients [5-7]. Recently, compelling studies suggests that while long-standing diabetes is an etiologic factor for pancreatic cancer, new-onset diabetes is caused by the cancer [5,8]. Importantly, new-onset diabetes is present in nearly half of all pancreatic cancer, and these patients display diabetes alongside paradoxical weight loss before the tumor is radiologically detectable [8]. These recent finding indicated a potential screening strategy for PC using manifestation of new-onset diabetes as an indicator of underlying pancreatic cancer. However, given that the primary type 2 diabetes and PC associated new-onset diabetes are still clinically indistinguishable, a reliable biomarker for PC associated new-onset diabetes remains to be identified before this screening strategy becomes cost-effective [8,9].

Understanding the mechanism of PC associated new-onset diabetes has broader implications for early diagnosis of pancreatic cancer, but the pathogenesis of PC-associated diabetes is still unknown. Current epidemiologic, clinical, and in vitro studies suggest that tumor-secreted products is more likely related toβ-cell dysfunction in PC associated diabetes rather a local tumor effect such as infiltration or obstruction [9,10]. Inflammation has been proposed to contribute to β -cell dysfunction in both type 1 diabetes (T1DM) and type 2 diabetes (T2DM) [11,12]. Indeed, islets from diabetes patients show immune cell infiltration and increased cytokine and chemokine expression [13]. Macrophage migration inhibitory factor (MIF) is a pro-inflammatory cytokine and an important regulator of innate immunity [14]. Although first described as an immune cell product, a much higher MIF level was found in kinds of human cancer and cancer-prone inflammatory diseases, including chronic pancreatitis and pancreatic cancer [14,15]. In addition, many functions of MIF support its potential involvement in diabetes, such as MIF inhibits INS-1 cell proliferation, MIF deficiency in atherosclerosis-prone mice impairs the development of insulin resistance, and MIF contributes to beta cell death during exposure to toxic nutrients, palmitic acid, or glucose [16,17]. Despite the compelling evidence suggesting MIF play role in cancer and diabetes, there is no data whether MIF could promote PC associated diabetes (PCDM) and distinguish it from T2DM.

In this study, we aimed to demonstrate the biological relevance of MIF in pancreatic cancer induced β-cell dysfunction, and to identify whether it could serve as a potential biomarker of PC associated diabetes that can distinguish it from type 2 diabetes.

## Materials and methods

### Cell culture

HIT-T15 pancreatic β-cells were kindly provided by the Endocrine laboratory of Sun Yat-sen memorial hospital, and were maintained in the RPMI 1640 (Gibco, Grand Island, NY) with 10% fetal bovine serum (FBS), 2 mmol/L glutamine, 100 IU/ml Penicillin and 100 mg/ml streptomycin. Human primary pancreatic cell lines were purchased from the American Type Culture Collection and grown in RPMI 1640 (Gibco, Grand Island, NY) and DMEM (Gibco, Grand Island, NY) with 10% FBS, 1% penicillin/streptomycin. HPDE6 (immortalized human pancreatic ductal epithelial cells) was obtained form Dr. Zhang SN (Sun Yat-Sen University, Guangdong, China).

### Islet isolation

For isolation of pancreatic islets, normal adult male inbred C57BL/6 mice were used, and 12-weeks old ones were sacrificed by cervical dislocation and their pancreas were removed. Islet cells were isolated from perfusion of the pancreas and digested using by collagenase V (Sigma-Aldrich, St Louis, MO) in Hank’s balanced salt solution followed by handpicking and placed in a 96-well plate (10 islet per well), grown overnight in RPMI 1640 (Gibco, Grand Island, NY) containing 10% FBS, 10 mmol/L HEPES, 5 μl/Lβ-mercaptoethanol, 2 mmol/L glutamine, 1 mmol/L Sodium Pyruvate, 100 IU/ml penicillin, and 100 m g/ml streptomycin in a humidified (5% CO2, 95% air) atmosphere at 37°C.

### RNA isolation and quantitative real-time PCR

Quantitative real time PCR (qPCR) was performed for MIF mRNA with GAPDH as an internal control. RNA was isolated from frozen pancreatic cancer tissues, chronic pancreatitis tissues, and pancreatic cell lines using the RNA easy spin mini-column kit (Qiagen Sciences, Valencia, CA) according to the manufacturer’s instructions, and total RNA was extracted. Total RNA was converted to cDNA by reverse-transcription using oligodT primers and SuperScript II reverse transcriptase (Invitrogen). For real-time quantitative PCR, three replicates of each sample were amplified and analyzed with a Roche Light-Cycler (Roche, Basel, Switzerland). Reactions were in a 20 μl volume by using SYBR Green reaction mix (Qiagen Sciences) with 0.5 mM of primer. Relative gene expression levels were determined using the Δ*C*t-method.

### MIF knock-in and knock-down

Lentiviral vectors were used to generating the stable MIF-over expressing and shRNA MIF-knockdown cells as described by Funamizu et al. Briefly, Lentiviral MIF knock-in constructs (pLOC-MIF), and lentiviral MIF knock-down constructs (pGIPZ-shRNA 1 and shRNA2) were obtained from Open Biosystems (Rockford, IL). Lentiviral particles were produced by transfecting 239 T cells according to the manufacturer’s protocol. Viral supernatants was collected after 72 hours following transfection, and the particles were concentrated by using LentiX^TM^ Concentrator over night at 4°C (Clontech, Mountain View, USA), the aliquots were stored at -80°C. The titers of the concentrated particles were measured before using. The PANC-1 and CAPAN-2 cells were (5× 10^5^cells/well)were seeded in 6-well culture plates, maintained in DMEM medium with 10% FBS for 24 hours before infection. For screening, blasticid in (10 μg/mL) for MIF Knock-in cells and puromycine (10 μg/mL) for MIF knock-down cells were added to the medium 72 hours after infection. The medium was replaced every 2 days for 2–3 weeks.

### Western blotting

Cells were washed in PBS and lysed with RIPA buffer (Invitrogen, Carlsbad, CA). For equal protein loading, a Bicinchoninic acid protein assay kit (Pierce) was used to calculate protein concentration in each sample. Equivalent amounts of proteins were separated by SDS-PAGE and transferred to polyvinylidene fluoride (PVDF) membranes for immunoblotting. The membranes were blocked in 5% fat-free milk for 2 hours at room temperature, washed 3 times, and then the membranes were incubated with the following primary antibodies: rabit anti-human MIF polyclonal antibody (1:1000, Abcam, Cambridge, MA), rabit anti-human β-actin antibody (1:1000, Abcam, Cambridge, MA), rabit anti-human GAPDH antibody (1:1000, Abcam, Cambridge, MA), anti-Src and anti-p-Src Tyr^418^ (1:500, Cell Signaling Technology, USA), and rabbit polyclonal to LCC α 1C (1:200; Alomone, Israel). β- actin or GAPDH was used for loading controls. Horseradish peroxidase-conjugated secondary antibodies (Cell signaling technology) and an ECL chemiluminescence kit (Pierce) were used to detect bound antibody.

### Immunohistochemistry

Paraffin-embedded samples of the primary carcinomas were stained for RASSF6. The pathological sections were deparaffinized in xylene and rehydrated in grade series of ethanols followed by heat-induced epitope retrieval in citrate buffer (PH = 6.0). Antigen retrieval was carried out in 10 mmol/l citrate buffer (pH = 6.0) in a microwave oven for 15 min. The activity of endogenous peroxidase was exhausted with 3% hydrogen peroxide for 10 min at room temperature. Rabbit MIF polyclonal antibody (Abcam, Cambridge, MA) were applied over night at 4°C at optimal working concentration of 1:300. After sufficient phosphate buffered saline rinses, sections were stained with goat anti-rabbit polymers.

### Voltage-dependent Ca2+ channel current recording

VDCC Ca2+ currents of HIT cell were monitored in a voltage-clamped HIT-T15 cell using the whole cell patch-clamp technique as previously described [18]. The extracellular recording solutions contained 140 mmol/L tetraethylammonium chloride, 5 mmol/L 4-aminopyridine, 5.4 mmol/L KCI, 1 mmol/L MgCI2, 2 mmol/LBaCI2, 2 mmol/L glucose, an d 10 mmol/L HEPES, and the pH was adjusted t o 7.3 with KOH. The pipette (intracellular solution) contained 105 mmol/L N-methylglucamine, 15 mmol/L 1,2-bis-aminophenoxyethane-N,N,N',N"tetra-acetic acid, 10 mmol/L HEPES, 2.5 mmol/L MgCI2, and 110 mmol/L L-aspartic acid, and the pH was adjusted to 7.2 with N-methylglucamine. MIF was also diluted to the appropriate concentration (0 mmol/L, 20 mmol/L, and 40 mmol/L) in the bath solution. The VDCC currents were recorded by a 300 ms step pulse ranging from -80 to +60 mV, the holding potentials were selected at -40 mV which represents mostly the activity of L-type VDCCs, and the out ward voltage-dependent K + currents or inward T-type VDCC currents were largely inactivated, respectively.

### Indirect co-culture of pancreatic cancer cells and β-cells or islets

HIT-T15 pancreatic β-cells (2 × 10^5^cells/well) were seeded in 12-well culture plates (Corning®) in modified PRIM 1640 medium supplemented with 10% FBS, 2 mmol/L glutamine, 100 IU/ml penicillin, 0.1 mg/ml streptomycin, and 5.5 mmol/L glucose. Isolated islets were dispersed into single cells by trypsin enzymatic digestion and gentle pipetting every 60s for 5 minutes, seeded in 12-well culture plates (cells of 20 islets per well), and then maintained in modified PRIM 1640 (Gibco, Grand Island, NY) supplemented with 10% FBS, 100 IU/ml penicillin, 0.1 mg/ml streptomycin, and 11 mmol/L glucose. Pancreatic cancer cells (2 × 10^5^cells/culture insert) were seeded into the transwell culture inserts of 1.0 μm pore size (Corning® BioCoat™) in PRIM medium supplemented with 10% FBS, penicillin, and streptomycin. After 24 hours, the culture insets were placed into the 12-well plates containing HIT-T15 cells, and incubation was continued up to 1 day in modified RPMI1640 medium supplemented with 1% FBS, glutamine, 5.6 mmol/L glucose, penicillin, and streptomycin.

### Insulin secretion assay

The effect of MIF on insulin secretion function was evaluated through two models: the indirect co-culture method, and the adding supernatants method. In the indirect co-culture method, both the HIT-T15 cells and the islets cells were co-cultured with PANC-1, Capan-2, or HPDE6 cells respectively in the 12-well transwell plates as mentioned above. 24 hours after co-culture, the medium was replaced with fresh modified KRB buffer (136 nM Na, 4.8 nM KCl, 2.5 nM CaCl2, 1.2 nM KH_2_PO_4_, 1.2 nM MgSO_4_, 5 nM NaHCO_3_, 10 nM HEPES, and 0.1% BSA, pH 7.3) containing 2.5 mmol/L glucose for 30 min at 37°C (basal secretion). And then, the KRB was removed from each well (collected for insulin assay, stored at -80°C) and replaced by the same modified KBR buffer containing 16.7 mmol/L glucose for 30 min (stimulatory secretion). At the end, this KBR was also collected and stored at -80°C. In the other method, the supernatants of PANC-1, Capan-2, or HPDE6 cells were added into the culture medium (the same modified PRIM1640 medium used in the co-cultured method) of HIT-T15 cells and islet cells, respectively. After 24 hours, the medium was replaced with fresh modified KRB buffer (136 nM Na, 4.8 nM KCl, 2.5 nM CaCl2, 1.2 nM KH_2_PO_4_, 1.2 nM MgSO_4_, 5 nM NaHCO_3_, 10 nM HEPES, and 0.1% BSA, pH 7.3) containing 2.5 mmol/L glucose and supplemented with or without the supernatants for 30 min at 37°C (basal secretion). And then, the KRB was also removed, collected, and replaced by the same modified KBR buffer containing 16.7 mmol/L glucose, supplemented with or without the supernatants, for 30 min at 37°C (stimulatory secretion). This KBR was also collected and stored at -80°C. At last, Insulin level was measured. HIT Cells and islets cells were lysed in RIPA buffer and total protein determined by BCA Protein Assay Kit (Pierce, USA). The insulin dose was normalized to cellular protein.

### Evaluation of serum MIF levels

The serum MIF levels were measured in 70 patients without PC and other malignant disease or immune disease, and in 105 patients diagnosed with pancreatic cancer. Informed consent was taken from all subjects. The Human Research Ethics Committee of Sun Yat-sen Memorial Hospital approved all aspects of this study. Blood samples were collected immediately after the patients was admitted to the hospital under fasting state at about 8 AM in a 5 ml dying vacuum tube, and then the serums were separated and collected after blood clotting, stored in liquid nitrogen. The levels of serum MIF were quantified using commercial ELISA kits according to the manufacturer’s instructions (R&D Systems, Minneapolis, MN, USA).

## Results

### MIF expression is associated with diabetes history in resected pancreatic cancer tissues

**Figure 1:**
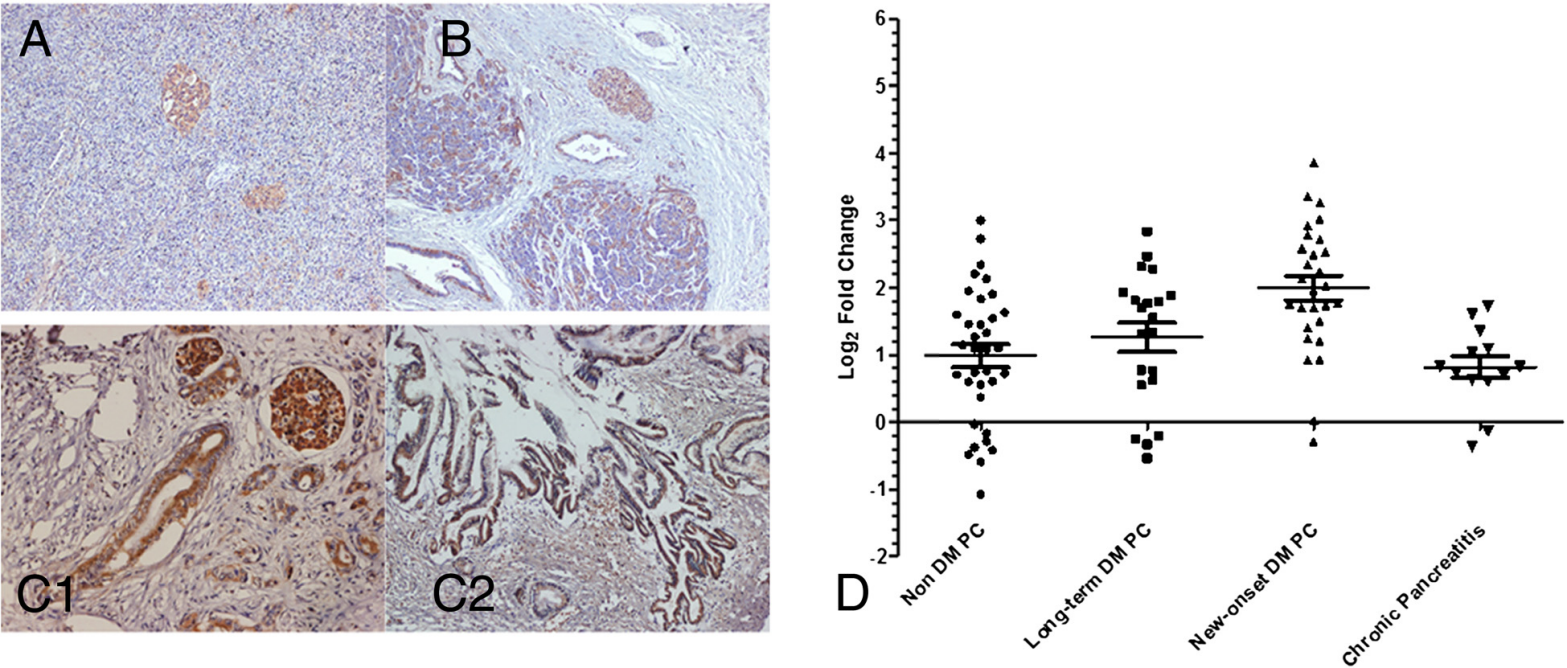
**MIF is overexpressed in human pancreatic cancer tissues, especially in those with diabetes, compared with PC patients without DM history or chronic pancreatitis patients.** Immunohistochemical analysis of MIF in paraffin. sections shows: **(A)** Chronic pancreatitis showing weak, sporadic cytoplasmic staining in gland, and weak to moderate, diffused cytoplasmic staining in islets (red arrow). **(B)** Normal pancreas, islets also showed weak to moderate MIF staining, but MIF expression in adjacent glands was much weaker and frequently lost. **(C1&C2)** In pancreatic cancer tissues, an increased, diffused cytoplasmic MIF expression in both tumor cells and adjacent islets (red arrow). **(D)** Real-time quantitative PCR on pancreatic cancer tissues form patients with different DM history and chronic pancreatitis tissues. Compared with the other groups, an significant increased MIF mRNA level in new-onset DM PC tissues was observed.

A total of 84 pancreatic cancer patients were included in the immunohistochemistry and qRT-PCR analysis (Additional file 1: Table S1 and Additional file 1: Table S2). On immunohistochemistry, in the normal pancreata (Figure 1A), the islets were moderate stained for MIF, while glands and ducts stained weakly. Chronic pancreatitis tissues showed a stronger MIF staining than normal pancreata both in islets, glands and ducts (Figure 1B). In comparison, pancreatic cancer specimens showed a strong positive MIF staining (Figure 1C); MIF expression was found not only in cancer cells but also neighboring ducts, acini, and islets. The islets adjacent to the cancer also showed a diffused over-expression pattern.

MIF gene expression analysis was performed in resected samples using qRT-PCR (Figure 1D). Quantitative RT-PCR on RNA isolated from human pancreatic cancer tissues showed higher MIF expression in patients with new-onset DM compared with patients with long-term DM or those without DM. Expression level of MIF gene was similar between the group of chronic pancreatitis and non-DM pancreatic cancer.

### MIF overexpression impairs insulin secretion

**Figure 2:**
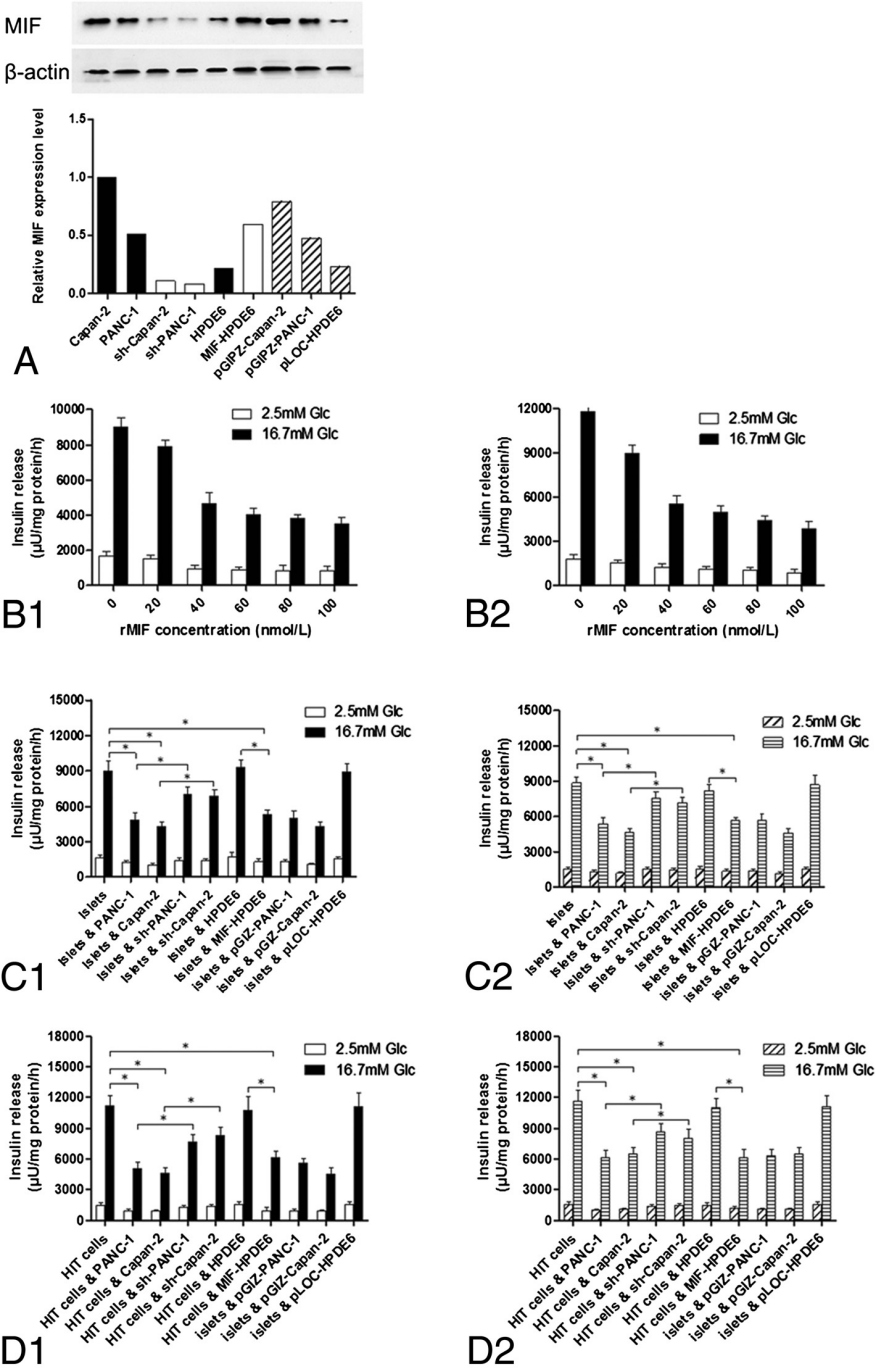
**MIF inhibits glucose-stimulated insulin secretion and contributes to the insulin inhibitory effect of PC cells. (A)** Stable shRNA MIF-knockdown (sh-Capan-2, sh-PANC-1) cells and MIF-over expressing cells (MIF-HPDE6) were generated by using lentiviral vectors. Vector controls showed no regulatory effect on MIF expression. **(B1&B2)** Degree of inhibitory effect on insulin releasing improved with the increasing concentration of MIF from 0 to 100 nmol/L both in PANC-1 **(B1)** and Capan-2 **(B2)** cell lines. A most significant drop of insulin secretion level was observed following exposure to MIF at concentrations of 40 nmol/L, reduced to 52% in islets and 46.7% in HIT-T15 cells, respectively, 0 nmol/L rMIF act as control groups. (P <0.01, n = 6). **(C1&C2)** MIF mediated the inhibitory effect of PC cells on insulin secretion capacity. **C1**: after indirectly co-cultured with PANC-1 or Capan-2 cells, islet cells showed impaired glucose-stimulated insulin secretion function, shRNA-lentivirus mediated MIF inhibition ameliorated the insulin secretion capacity; the immortalized epithelial cell line HPDE6 decreased insulin secretion a little, while MIF-transgene expressing HPDE6 cells showed a strong inhibitory effect on insulin secretion. **C2**: after adding supernatants into the culture medium of islet cells, a same effect of MIF was observed. **(D1&D2)** The same role of MIF was observed in HIT-T15 cells just as it shown in the islet cells. Glc: glucose; *:p<0.01, Student’s t-test. Sh-PANC-1: shRNA MIF-knockdown PANC-1 cell line; Sh-Capan-2: shRNA MIF-knockdown Capan-2 cell line; MIF-HPDE6: MIF-transgene overexpression HPDE6 cell line; pGIZ-PANC-1, pGIZ-Capan-2, and pLOC-HPDE6 are null vector transfected cell lines as controls to exclude the effect of vector on expression of potential genes coding proteins which might regulate insulin releasing.

Because MIF was overexpressed in pancreatic cancer cells, we used MIF shRNA to knock down MIF in PANC-1 and Capan-2 cells. Vice versa, a MIF overexpressing immortalized human pancreatic ductal epithelial cell line was generated. The MIF expression was analyzed by western blot and real-time PCR, results showed that stable MIF-over expressing immortalized human pancreatic ductal epithelial cells (HPDE6) and shRNA MIF-knockdown pancreatic cancer cells (PANC-1 and Capan-2) were generated (Figure 2A).

The diabetogenic effect of MIF on glucose-stimulated insulin secretion from HIT cells and isolated mouse islets was studied first through adding rMIF into the medium directly. As shown in Figure 2B, we observed a dose–response relationship between MIF concentration and the inhibiting effect on insulin secretion. When islets cells (Figure 2B1) or HIT cells (Figure 2B2) were stimulated with 16.7 mmol/L glucose following exposure to MIF at concentrations of 40 nmol/L, insulin secretion was reduced to 52% and 46.7%, respectively (P <0.01, n = 6).

After indirectly co-cultured with PANC-1 or Capan-2 cells, or treated with PANC-1/Capan-2 supernatants (volume of original medium of insulin secreting cells: volume of supernatants is about 1:1), both islet cells and HIT cells showed impaired glucose-stimulated (16.7 mmol/L) insulin secretion function, and shRNA-mediated knockdown of MIF ameliorated the inhibitory effect of PANC-1 and Capan-2 cells on insulin secretion capacity. The immortalized epithelial cell line HPDE6 had little impact on insulin secretion, while MIF-transgene expressing HPDE6 cells or its supernatants showed a strong inhibitory effect on insulin secretion (Figure 2C and Figure 2D).

### rMIF stimulation depresses HIT cell VDCC Ca2 + currents

**Figure 3:**
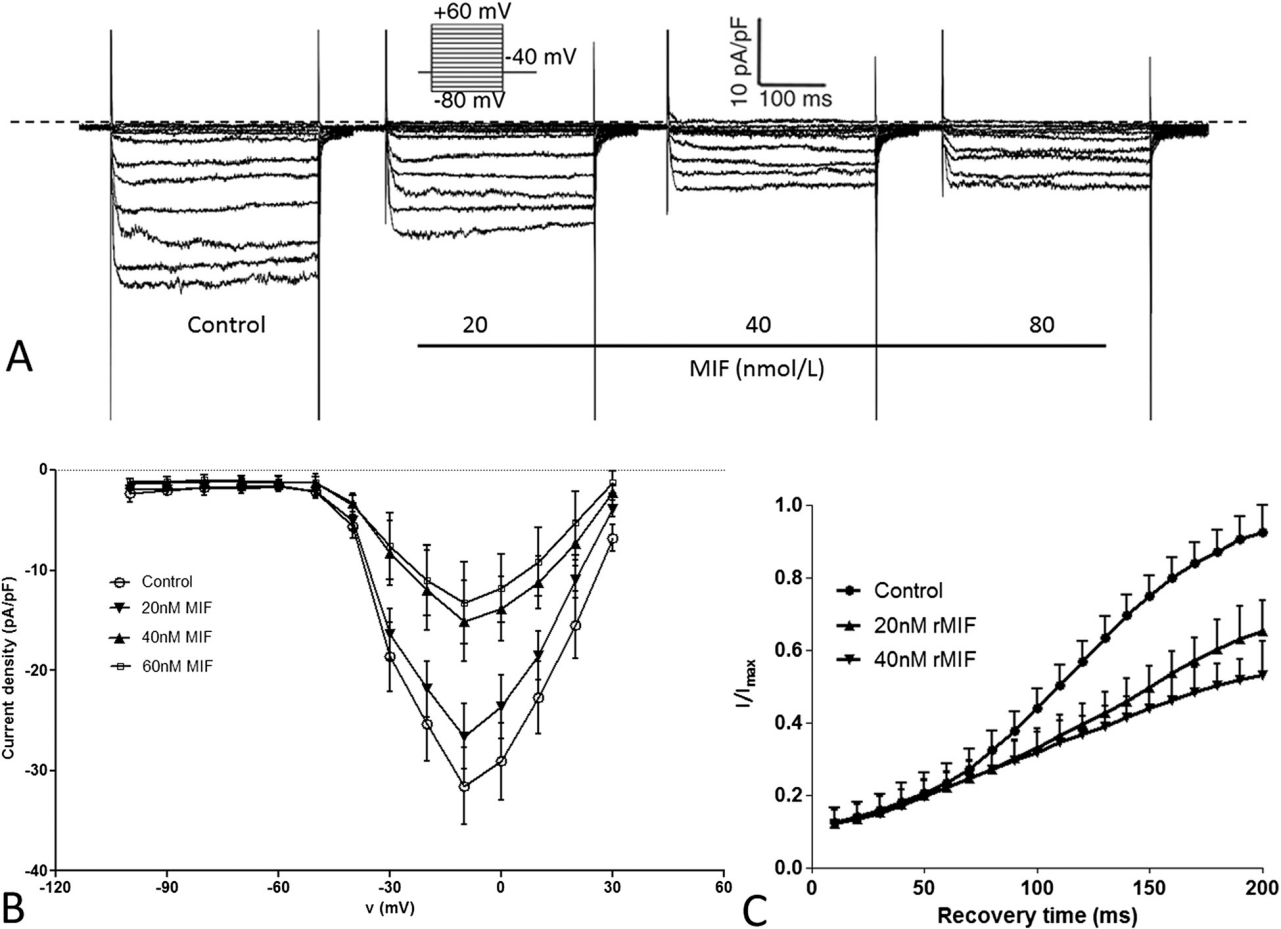
**The inhibitor effect of MIF on L-type voltage-dependent Ca2+ channels in mouse pancreatic β cells (HIT-T15 cell line). (A)** Representative recordings of whole-cell ICa,L are shown for the control cells, and cells treated with 20 nM, 40 nM, or 60 nM rMIF for 24 h. **(B)** Representative ICa,L density– voltage relationships in control cells (n = 6) and cells treated with 20 nM (n = 6), 40 nM, or 60 nM rMIF (n = 6). **(C)** A double-pulse protocol is shown with the testing potentials stepped from - 70 to 0 mV at an HP of - 40 mV. The interval between two pulses was 10 ms. An inhibitory effect of MIF on the recovery kinetics from L-type voltage-dependent Ca2+ channels inactivation was observed.

Cultured HIT cells were pretreated with 20, 40 or 60 nmol/L rMIF for 24 h, and whole-cell evoked Ca2+ currents (L-type Ca 2+ currents, ICa,L) were recorded, and the current densities were calculated (n = 6). As shown in Figure 3A and 3B, on treatment with 20, 40, or 60 nmol/L rMIF, the ICa,L of HIT-T15 cell were depressed, and the peak current density of ICa,L was reduced, with reductions of 16%, 52% and 57%, respectively. The peak inward current densities fell from -31.6 ± 3.9 pA/pF in controls (n = 6) to -26.6 ± 3.3 pA/pF (20 nM, P < 0.05), to -15.1 ± 4.0 pA/pF (40 nM, P < 0.01), to -13.2 ± 4.1 pA/pF (60 nM, P < 0.01).

In order to evaluate the recovery from inactivation, currents were elicited by the double-pulse protocol with an interval separating two pulses increased from 0 to 200 ms. The time constants (τ) of ICa,L recovery from inactivation in the HP (Holding potential) of -40 mV were 96.2 ± 7.9 ms, in the controls (n = 3), significantly less than 136.7 ± 9.7 ms (n = 3), and 165.36 ± 12.9 ms (n = 3) in the presence of 20 nmol/L or 60 nmol/L rMIF, respectively. In addition, the I/Imax values in the rMIF-treated groups were significantly decreased in comparison with those of the control group (n = 3, P < 0.01) (Figure 3C). These results suggest that rMIF also impairs L-type Ca2+ channel (LCC) function through inhibiting the recovery speed of ICa,L from inactivation.

### rMIF down-regulates the expression of channel protein

**Figure 4:**
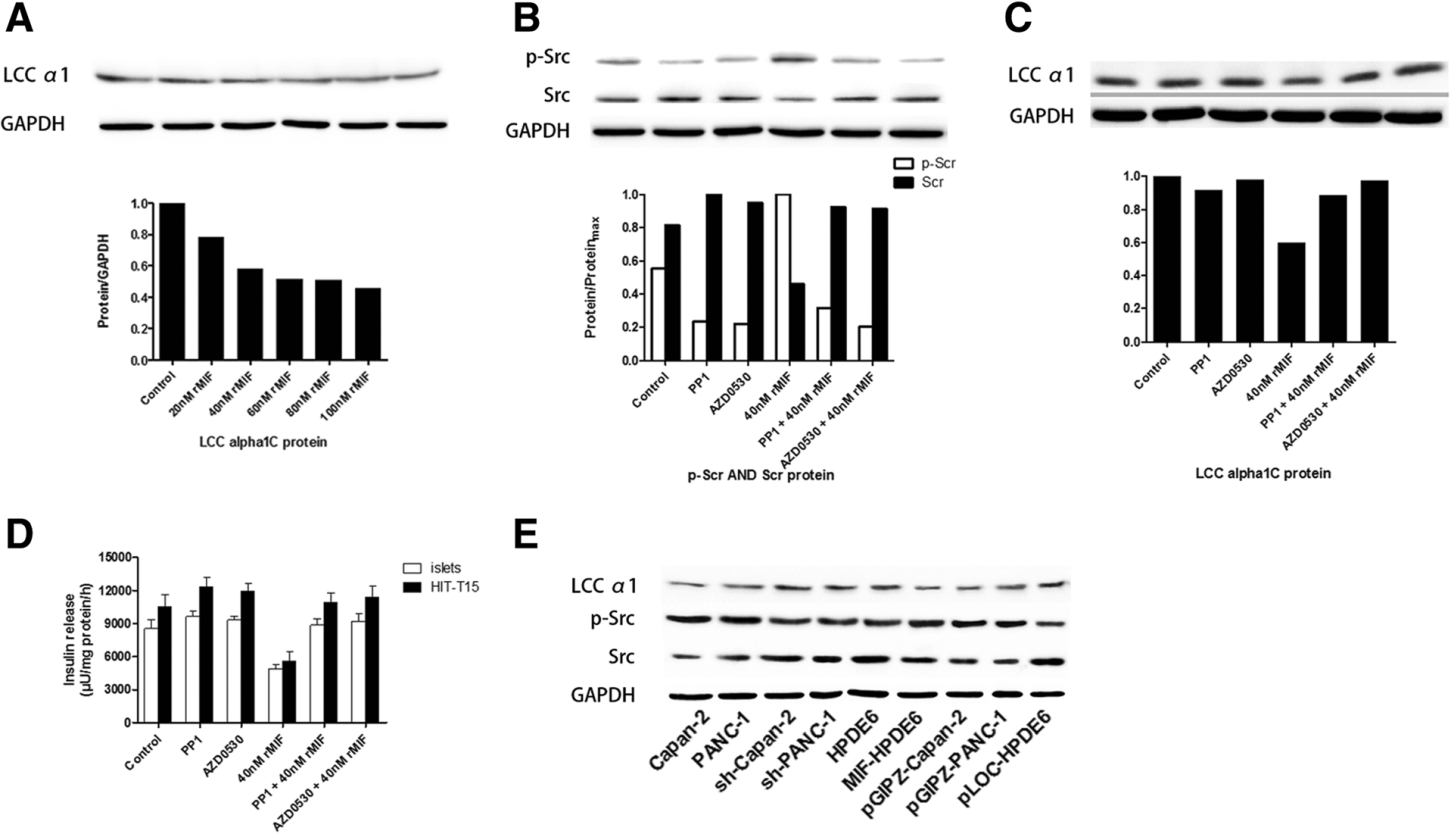
**Effect of MIF on LCC α1C subunit protein and Src activity in HIT-T15 cells. (A)** Expression of LCC α1C subunit in HIT-T15 cells was decreased following rMIF-treatment in a concentration dependent manner. 40 nmol/L rMIF treatment showed the most obvious suppression effect on LCC α1C subunit expression. Top: representative Western blot analysis of LCC α1C protein in treated cells and densitometric analysis of Western blot analysis result. Bottom: LCC α1C subunit mRNA level was also decreased along with rMIF treatment. **(B)** Protein tyrosine kinase inhibitors antagonized the effect of MIF on Scr activity. Top: representative Western blot analysis of Scr activity in treated cells and controls; GAPDH was the internal control. Bottom: densitometric analysis of Src protein in treated cells and controls. **(C)** Protein tyrosine kinase inhibitors antagonized the effect of MIF on down-regulating LCC α1C protein level. Top: representative Western blot analysis of LCC α1C protein in treated cells; GAPDH was the internal control. Bottom: densitometric analysis of LCC α1C protein in treated cells and controls. **(D)** Insulin secretion assay on islets and HIT-T15 cells treated with rMIF with or without co-treatment of Src inhibitors. The inhibitory effect of rMIF on insulin secretion was eliminated by Src inhibitors. **(E)** In the indirect co-culture method of islets with PC cell lines, LCC α1C subunit expression and Src activity was evaluated through Western blot analysis. Results showed that MIF mediated the regulatory effect of PC cell lines on islets, the LCC α1C subunit expression level was in consistent with Src activity. Sh-PANC-1: shRNA MIF-knockdown PANC-1 cell line; Sh-Capan-2: shRNA MIF-knockdown Capan-2 cell line; MIF-HPDE6: MIF-transgene overexpression HPDE6 cell line; pGIZ-PANC-1, pGIZ-Capan-2, and pLOC-HPDE6 are null vector transfected cell lines as controls to exclude the effect of vector on expression of potential genes coding proteins which might regulate insulin releasing.

The LCC density was also associated with insulin secretion function. Therefore, to evaluate if MIF can affect channel expression level, we evaluated the expression of LCC α1 subunit before and after rMIF treatment. A dose-effect relationship was observed (Figure 4A), data revealed that the LCC α1 protein level gradually reduced with the increase of rMIF concentration. Meanwhile, cells pretreated with 20 and 40 nmol/L rMIF showed a more obvious extent of reduction in LCC α1 expression level compared with those treated with 60, 80 and 100 nmol/L rMIF, which was in consistent with the findings in the insulin secretion experiment. Quantification of western blot results revealed that α1C subunit protein level of HIT cells dropped 22% (P < 0.05) and 42% (P < 0.01) after treatment with 20 and 40 nmol/L rMIF, respectively. There was no statically difference in α1Csubunit protein level between cells receiving 60, 80, and 100 nmol/L rMIF pretreatment, and cells receiving 100 nmol/L rMIF pretreatment showed a slightly decreased viability (data not shown). Which indicate that 40-60 nmol/L rMIF might cause maxium efficiency on regulating α1C subunit protein level. The α1C subunit mRNA levels were also decreased after rMIF treatment (Figure 4A). Also, 20 and 40 nmol/L rMIF treatment showed more efficient impact on down-regulating α1C subunit mRNA (felled 30% and 39%, respectively), and concentrations higher than 40 nmol/L showed no further effect on regulating α1C mRNA.

### rMIF-induced β cell dysfunction is partially mediated by regulating Src family tyrosine kinase acitvity

Because it has been reported that the Src exert a tonic inhibitory role on Ca2+ dependent insulin secretion, the next series of experiments was performed to determine whether the effect of MIF on insulin secretion and Ca2+ channel protein expression were through regulating the Src activity. We found that 40 nmol/L rMIF treatment markly increased the level of p-Src (p < 0.01, n = 3), and both 15 μM PP1 and 2 μM AZD0530 almost entirely counteracted rMIF induced Src phosphorylation as expected (p < 0.01, n = 3) (Figure 4B).

Next, the effect of PP1 and AZD0530 on α1C subunit level was tested (Figure 4C). We found there was no significant change in α1C protein and mRNA levels when HIT-T15 cells were incubated with 15 μM PP1 or 2 μM AZD0530 for 24 h (GAPDH was internal control for western blot, β-actin was internal control for qRT-PCR, 0.93 ± 0.06 and 0.91 ± 0.05, respectively), but levels of α1C subunit protein were significantly higher in HIT-T15 cells treated with rMIF plus PP1 or AZD0530 in comparison with cells treated with rMIF along (p < 0.01, n = 3, both). This finding reflect that Src family kinase is likely to be involved in the rMIF-induced α1C down regulation. In addition, the diabetogenic effect of 40 nmol/L rMIF treatment on glucose-stimulated (16.7 mmol/L) insulin secretion function of both HIT-T15 cells and islet cells was also reversed through co-treatment with 15 nM PP1 or 2 nM AZD0530 (p<0.01, n = 6, both) (Figure 4D).

Since the HIT-T15 cell is a rat-derived insulin-secreting cell line, and some aspects of protein expression and exocytotic characteristics differ between this cell line and islets, we further confirmed the findings in pancreatic cancer cell line and isolated islet cells. As shown in Figure 4E, the α1C protein was down-regulated and the p-Src was up-regulated through co-cultured with PANC-1 and Capan-2 cells. Following shRNA-mediated knockdown of MIF, the regulatory effect of PANC1 and Capan-2 cells on α1C protein expression and Src activity was abolished. Vice versa, MIF-transgene expressing enabled HPDE6 cells to reduce α1C protein expression and to increase p-Src level. On the aspect of insulin secretion, the regulatory effect of PANC1, Capan-2, and MIF-expressing HPED6 cells on insulin secretion from islet cells was also significantly ameliorated by using PP1 or AZD0530 (131% vs. 59%, P<0.01, n = 6; 127% vs. 53%, P<0.01, n = 6; 133% vs. 60%, P<0.01, n = 6; respectively) (Figure 4E).

### MIF is a candidate serum biomarker of new-onset diabetes associated pancreatic cancer

**Figure 5:**
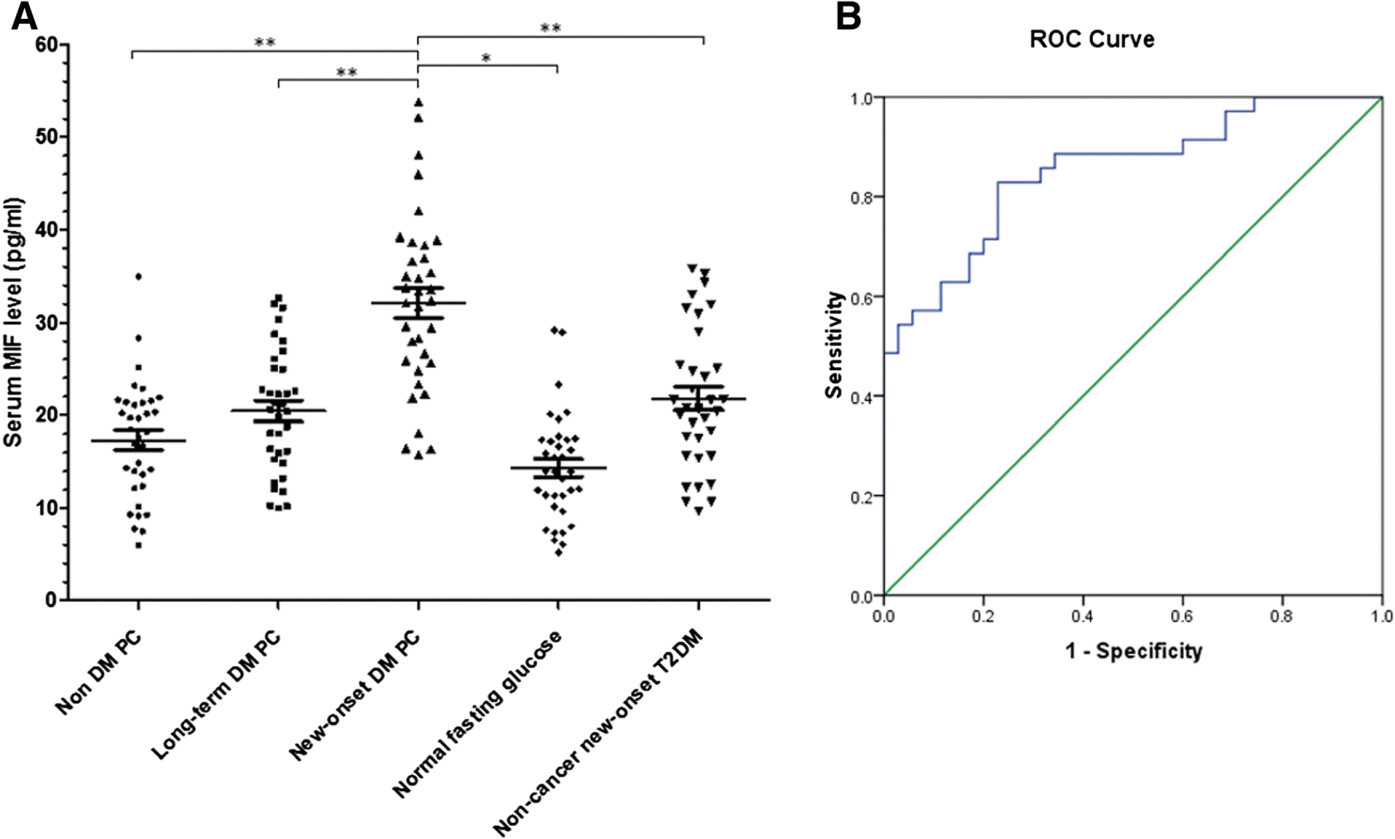
**MIF is a potential biomarker of PCDM. (A)** New-onset DM PC patients had the highest level of serum MIF level. **(B)** The receiver operating characteristic curve (ROC) revealed a sensitivity of 86% and a specificity of 60% to distinguish new-onset DM PC cases from new-onset T2DM patients. *:p<0.01; **:p<0.001; Student’s t-test.

As shown in Figure 5, the serum levels of MIF in the following five group patients were examined: pancreatic cancer patients without diabetes, pancreatic patients with more than two years history of diabetes, pancreatic patients with less than two years history of diabetes (new-onset diabetes), healthy subjects with normal fasting glucose, and T2DM patients (without any other immune diseases or tumor) diagnosed within in two years. The five groups were age (±3 years) and sex-matched, and the TNM stage of three pancreatic cancer groups are not significant different (Additional file 1: Table S3 and Additional file 1: Table S4). A scatterplot of MIF levels in the 5 groups is shown in Figure 5A. Mean plasma levels (ng/ml) were significant higher in 35 new-onset DM PC patients (32.1 ± 9.6) compared with 35 non-DM PC patients (17.3 ± 6.4, P <0.001), 35 long-term DM PC patients (20.4 ± 6.6, P <0.001), 35 normal fasting glucose healthy people (14.3 ± 5.8, P < 0.001), and 35 new-onset T2DM patients (21.8 ± 7.57, P <0.01). The area under the ROC (receiver operating characteristic curve) (Figure 5B) was 0.852, with an optimal cut off point of 18.1/ml. MIF had a sensitivity of 86% and a specificity of 60% to distinguish new-onset DM PC cases from new-onset T2DM patients.

## Discussion

Prognosis of pancreatic cancer is extremely poor, early detection of pancreatic cancer appears to provide the greatest prospect to improving long-term survival. However, screening in the general population is now high-cost and difficult since there is no ideal markers. Therefore, to identify the high risk population and biomarkers of pancreatic cancer is urgently to be addressed.

The association between pancreatic cancer and diabetes has long been studied for hundreds of years [19]. Now compelling studies suggest that long-term diabetes is an etiologic factor for pancreatic cancer, while new-onset diabetes is its early clinical manifestations, thus the new-onset diabetes of pancreatic cancer patients is consider as pancreatic associated diabetes (PCDM) [9,19,20]. It is notable that PCDM has both similarities and differences compared with the common type 2 DM. Common T2DM is usually associated with the metabolic syndrome, weight gain and family history of diabetes. In contrast, PCDM is often present in patients without family history, and hold manifestation of weigh lost [21]. Which indicate that the pathological mechanism of PCDM is different from that of T2DM. Although the pathogenesis of PCDM remains unclear, it gathered much more attention because such understanding might contribute to discover biomarkers for the early diagnosis of pancreatic cancer.

Macrophage migration inhibitory factor (MIF) is a pro-inflammatory cytokine involved in many inflammatory reactions, and it has become evident that it also affects glucose homeostasis through various pathways [16]. Since MIF showed a dual regulation activity on inflammatory and metabolic effects, it represents a logical focus of study in the field of diabetes. In the present study, we show that MIF is overexpressed in human PC tissue, especially in tissues of patients with new-onset diabetes, and is found elevated in the blood of new-onset DM PC patients. Through the experiments, we identify that MIF impairs insulin secretion activity of beta cell lines as well as isolated rat pancreatic islets through regulating Ca2+ channels function. Our find strongly suggest that MIF is a candidate biomarker of new-onset DM PC.

Under physiological conditions, MIF is produced by pancreatic β cells and can maintain its insulin secretion activity [22-27], but scholars became interested in its role in kinds of glucose metabolic disorder in recent years, including T1DM, T2DM, gestational diabetes mellitus (GDM), and obesity induced insulin resistance (IR) [16,28,29]. Several clinical and basic evidences support a relationship MIF level and diabetes. For example, the expression of MIF is increased in both pancreatic beta cells and peripheral lymphocytes of mice with autoimmune diabetes, in addition, in NOD mice treated with rMIF the incidence of diabetes is increased from 55% to 86% [30,31]. MIF-KO mice demonstrated an age-dependent weight gain, together with impaired both blood glucose and insulin secretion function, which seems like a T2DM pattern [32]. Serum concentrations of MIF are elevated in patients with T2DM in comparison with patients with impaired glucose tolerance and healthy individuals [33-36]. A significantly elevated serum MIF level in patients with GDM compared with healthy pregnant was also observed in a prospect study [37]. These reports are in consist with our findings in this study that the expression level are both increased in tissues and serum of patients with DM history, and support that increased MIF expression are associated with new-onset DM PC.

Actually, MIF might be involved in diabetes pathogenesis in much more ways: regulation the insulin secretion, modulation inflammation such as exacerbates insulitis and local pancreatic inflammation, contribute to beta cell apoptosis in cooperate with other toxics [15,17,30,31,38-41]. Because this present study is mainly intended to supply clinical evidence for screening PC, we did not dig the mechanism of the pro-diabetic effect of MIF too much, and just explored the influence of MIF on Ca2+ channels function and Src activity of beta cells. Since there are convincing evidences that insulin secretion function of islet cells is dependent largely on both L-type voltage-dependent Ca2+ channels and Src activity, our basic research findings provided reasonable support and explanation for the clinical data. Although MIF was reported to influence the validity of beta cells, we just observed a slight pro-apoptotic effect of rMIF on islet cells (8.3%) and beta cells (7.5%) in high concentration (80 nmol/L rMIF). This paradox may be explained by that its pro-apoptotic effect depends upon cooperation with other pro-inflammatory cytokines and toxics. Similarly, Saksida T et al. also reportedβ-cells treated with MIF along did not demonstrate a significant apoptosis rate [15].

Importantly, serum MIF level was reported to be associated with gender. In a large case-cohort study, elevated serum MIF level was associated with an increased risk of T2DM in female but not in male [34]. The reported difference between serums MIF level in male and in female with T2DM might linked to single nucleotide polymorphisms and the influence of sex hormones [42]. Thus, in our present study, the long-standing DM PC cases was choose in a same sex ratio with new-onset PC patients, and then the controls (normal fasting glucose group, and new-onset T2DM group) was matched to the cases (new-onset PC group) with both age and sex in order to avoid bias. Interestingly, in the non-cancer new-onset T2DM group, the serum MIF levels of female seemed to be higher than that of male (p = 0.051), but there was no difference in serum levels of MIF between male and female patients with new-onset DM PC (p = 0.362). This result is probably explained by that the onset of T2DM is more relevant to genetic backgrounds and systematic disorder, but the pathogenesis of PCDM is likely to be a humoral process which impairs β cell function and peripheral insulin resistance induced by tumor itself [8,9].

## Conclusions

The pathogenesis mechanism of PCDM is only starting to be uncovered, but such understanding might yield biomarkers which can distinguish PCDM from the more common new-onset T2DM. In this study, we elucidated that MIF impairs glucose-stimulated insulin secretion of β-cells through inhibiting Ca2+ channels and regulating Src family tyrosine kinase activity. Our study is one of the few researches on the diabetogenic ability of pancreatic cancer cells, and further studies to investigate the role of MIF as well as identify other candidate mediators of PCDM are needed.

## Abbreviations

PC: Pancreatic cancer; DM: Diabetes mellitus; MIF: Macrophage migration inhibitory factor; rMIF: Recombinant macrophage migration inhibitory factor; rMIF: ancreatic cancer associated diabetes mellitus; T1DM: Type 1 diabetes mellitus; T2DM: Type 2 diabetes mellitus; GDM: Gestational diabetes mellitus; VDCC: Voltage-dependent Ca2+ channel; L-VDCC: L-type Voltage-dependent Ca2+ channel; LCC: L-type Ca2+ channel; LCCα1: L-type Ca2+ channel subunit α1.

## Competing interests

The authors declare that they have no competing interests.

## Authors’ contributions

RFC and QBZ conceived of and carried out experiments, analysed and interpreted data and drafted the manuscript; LPT and YX conceived of experiments, analysed and interpreted data and wrote the manuscript; YZ, MY, FZQ, RWC, BXZ, YHL, QL, and WCG analysed and interpreted data. All authors read and approval the final manuscript.

## Supplementary Material

Additional file 1: Table S1 Number and TNM stage of patients included in immunohistochemical and PCR analysis. **Table S2.** Chi-square test (Fisher’s exact test) for the AJCC stage of different groups. **Table S3.** Number and TNM stage of patients included in evaluation of blood MIF levels. **Table S4.** Chi-square test (Fisher’s exact test) for the AJCC stage of different groups of **Table S3.** Click here for file
