# Supplementary material for: Macrophage migration inhibitory factor is overexpressed in pancreatic cancer tissues and impairs insulin secretion function of β-cell
Source: J Transl Med. 2014 Apr 7;12:92. doi: 10.1186/1479-5876-12-92 (PMC4022046; doi:10.1186/1479-5876-12-92)
Supplement: Additional file 1: Table S1 — Number and TNM stage of patients included in immunohistochemical and PCR analysis. Table S2. Chi-square test (Fisher’s exact test) for the AJCC stage of different groups. Table S3. Number and TNM stage of patients included in evaluation of blood MIF levels. Table S4. Chi-square test (Fisher’s exact test) for the AJCC stage of different groups of Table S3. [file 1479-5876-12-92-S1.docx]

**Table S1.** Number and TNM stage of patients included in immunohistochemical and PCR analysis.

| **Gourp of patients** | **Number of patients** | **Gender**  **male, female** | **Age**  **Median (Range)** | **AJCC stage** | |
| --- | --- | --- | --- | --- | --- |
|  |  |  |  | IB | IIA |
| Non DM PC | 35 | 20, 15 | 55 (39-71) | 8 | 27 |
| Long-term DM PC | 21 | 14, 7 | 51 (51-66) | 6 | 15 |
| New-onset DM PC | 28 | 17,11 | 49 (39-65) | 8 | 20 |

Non DM PC: pancreatic cancer patients without diabetes history or impaired fasting glucose

Long-term MDPC: pancreatic cancer patients with more than 2-years history of diabetes when first diagnosis

New-onset DM PC: pancreatic cancer patients with less than 2-years history of diabetes when first diagnosis

IB: T2 N0 M0

IIA:T3 N0 M0

**Table S2.** Chi-square test (Fisher’s exact test) for the AJCC stage of different groups.

| **Gourp of patients** | **Number of patients** | **AJCC stage** | | ***P* value*** |
| --- | --- | --- | --- | --- |
|  |  | **IB** | **IIA** |  |
| Non DM PC | 35 | 8 | 27 | 0.752 |
| Long-term DM PC | 21 | 6 | 15 |  |
|  |  |  |  |  |
| Non DM PC | 35 | 8 | 27 | 0.772 |
| New-onset DM PC | 28 | 8 | 20 |  |
|  |  |  |  |  |
| Long-term DM PC | 21 | 6 | 15 | 1.000 |
| New-onset DM PC | 28 | 8 | 20 |  |

*:Fisher’s exact test.

**Table S3.** Number and TNM stage of patients included in evaluation of blood MIF levels

| **Gourp of patients** | **Number of patients** | **Age, Median (range)** | **Gender and AJCC stage** | |
| --- | --- | --- | --- | --- |
|  |  |  | Male (AJCC IB: IIA) | Female (AJCC IB: IIA) |
| Non DM PC | 35 | 55 (39-71) | 20 (4:16) | 15 (4:11) |
|  |  |  |  |  |
| Long-term DM PC | 35 | 53 (41-71) | 20 (5:15) | 15 (4: 11) |
|  |  |  |  |  |
| New-onset DM PC | 35 | 53 (39-73) | 20 (6:14) | 15 (5:10) |

**Table S4.** Chi-square test (Fisher’s exact test) for the AJCC stage of different groups of Table 3.

| **Gourp of patients** | **Number of patients** | **AJCC stage** | | ***P* value*** |
| --- | --- | --- | --- | --- |
|  |  | **IB** | **IIA** |  |
| Non DM PC | 35 | 8 | 27 | 1.000 |
| Long-term DM PC | 35 | 9 | 26 |  |
|  |  |  |  |  |
| Non DM PC | 35 | 8 | 27 | 0.592 |
| New-onset DM PC | 35 | 11 | 24 |  |
|  |  |  |  |  |
| Long-term DM PC | 35 | 9 | 26 | 0.785 |
| New-onset DM PC | 35 | 11 | 24 |  |

*:Fisher’s exact test.
